# Body size is negatively correlated with trophic position among cyprinids

**DOI:** 10.1098/rsos.150652

**Published:** 2016-05-11

**Authors:** Edward D. Burress, Jordan M. Holcomb, Karine Orlandi Bonato, Jonathan W. Armbruster

**Affiliations:** 1 Department of Biological Sciences and Museum of Natural History, Auburn University, 101 Life Sciences Building, Auburn, AL 36849, USA; 2 Florida Fish and Wildlife Conservation Commission, Fish and Wildlife Research Institute, 7386 Northwest 71st Street, Gainesville, FL 32653, USA; 3 Departamento de Zoologia, Universidade Federal do Rio Grande do Sul, Instituto de Biociências, CEP 91501-970, Porto Alegre, Rio Grande do Sul, Brazil

**Keywords:** benthic–pelagic axis, community structure, Cyprinidae, diet, coevolution

## Abstract

Body size has many ecological and evolutionary implications that extend across multiple levels of organization. Body size is often positively correlated with species traits such as metabolism, prey size and trophic position (TP) due to physiological and mechanical constraints. We used stable isotope analysis to quantify TP among minnows across multiple assemblages that differed in their species composition, diversity and food web structure. Body size significantly predicted TP across different lineages and assemblages, and indicated a significant negative relationship. The observed negative relationship between body size and TP is contrary to conventional knowledge, and is likely to have arisen owing to highly clade-specific patterns, such that clades consist of either large benthic species or small pelagic species. Cyprinids probably subvert the physiological and mechanical constraints that generally produce a positive relationship between body size and TP using anatomical modifications and by consuming small-bodied prey, respectively. The need for herbivorous cyprinids to digest cellulose-rich foods probably selected for larger bodies to accommodate longer intestinal tracts and thereby to facilitate digestion of nutrient-poor resources, such as algae. Therefore, body size and TP are likely to have coevolved in cyprinids in association with specialization along the benthic to pelagic resource axis.

## 1. Background

Body size is a fundamental characteristic of an organism, and it has myriad implications for behaviour, metabolism, population dynamics, community structure and evolution. Body size affects behavioural traits such as locomotion, habitat use and prey exploitation [1]. For example, consumer body size is positively correlated with prey size [2,3], which is probably due to either relaxed gape limitations or increased metabolic demands associated with larger body sizes [4]. Many metabolic factors are influenced by body size such as growth, reproduction and respiration [5,6]. Body size also affects population characteristics such as density and abundance [7,8] as well as community characteristics such as food web structure and food chain length [9].

In a broad survey of fish body size and trophic position (TP), Romanuk *et al*. [10] found that 26 of 57 orders of fishes exhibited a significant positive relationship, whereas none showed a negative correlation. A combination of physiological and mechanical constraints probably produces this relationship. For example, larger bodies have higher energy demands such that large-bodied organisms must consume nutrient-rich prey [4]. Additionally, with increasing body size, relaxed gape limitation allows for the consumption of large-bodied prey such as animals rather than plants. Here, we test the relationship between body size and TP among eastern North American cyprinids (Teleostei: Cyprinidae) across several stream assemblages. Cyprinids present conditions in which a novel, negative relationship may exist. For example, many benthic-oriented, omnivorous clades are larger bodied than pelagic-oriented, carnivorous clades, and top cyprinid predators are generally drift feeding insectivores rather than piscivores [11,12]. Cyprinids are ubiquitous in North American streams and often dominate stream communities in terms of abundance and species diversity [11,13]. Cyprinids have also frequently diversified along the benthic to pelagic axis [12,14] and thus exploit a variety of resources associated with the substrate, water column and water surface. Therefore, cyprinids provide a group that may exhibit variable TPs. In this study, we evaluate the relationship between body size and TP in five diverse cyprinid assemblages across four distinct river drainages.

## 2. Material and methods

**Figure 1.**
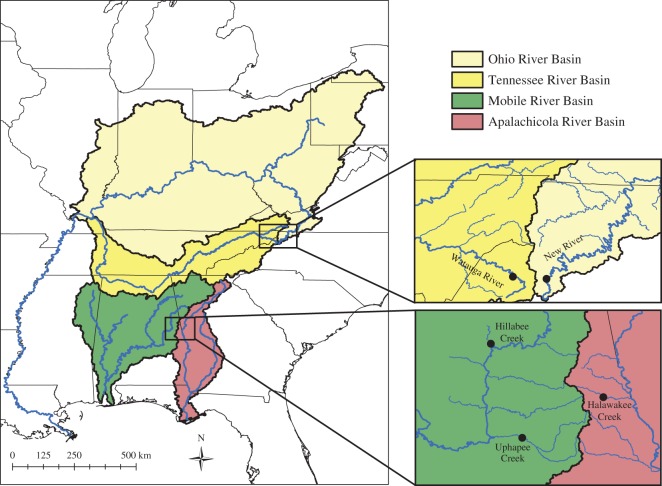
Map of the eastern continental USA. Inset boxes depict the locations of sampling localities: New River, Watauga River, Hillabee Creek, Uphapee Creek and Halawakee Creek.

We sampled five cyprinid assemblages (figure 1): the Watauga River and New River (Tennessee and Ohio river basins) in western North Carolina in March and July 2012, and Uphapee Creek and Hillabee Creek (Mobile River Basin) and Halawakee Creek (Apalachicola River Basin) in southeastern Alabama in November 2014 and February 2015, using a combination of seine and backpack Electro-fisher (Smith-Root, Inc.). The Watauga and New rivers are high gradient mountain streams with rocky substrate. Therefore, these cyprinid assemblages consist of species adapted to cold, well-oxygenated and high velocity conditions [15]. Hillabee and Halawakee creeks are low gradient piedmont streams with mostly rocky substrates. Uphapee Creek is a low gradient coastal plain stream and has mostly sand substrate. Therefore, the Alabama cyprinid assemblages consist of species adapted to warm, low velocity conditions, including many species (i.e. *Notropis*) that prefer sandy habitats [11]. Despite the close geographical proximity of the two North Carolina rivers and the three Alabama creeks that were sampled (figure 1), they have different assemblages associated with the four major river basins in which they drain, and thus represent a robust sampling of the major cyprinid lineages found in the eastern USA [12]. All species that we sampled (table 1), except *Semotilus* and *Clinostomus*, are part of a strongly supported clade united by the osteological character of a small opening at the base of the skull known as the open posterior myodome (OPM).

**Table 1.** Data used to assess the relationship between body size and TP among cyprinids: guild designation, relative gut length (RGL), body size (SL; cm) and TP.

sample	*n*	site	guild	RGL	SL	TP	^15^N	^13^C
*Rhinichthys cataractae*	8	new	benthic	0.92	5.8 ± 1.7	2.30	10.8 ± 0.4	−21.8 ± 1.1
*Luxilus coccogenis*	3	new	pelagic	0.60	7.2 ± 0.3	1.78	9.5 ± 0.2	−22.1 ± 0.2
*Semotilis atromaculatus*	5	new	benthic	0.94	7.9 ± 1.9	1.63	9.1 ± 0.8	−23.9 ± 0.1
*Notropis photogenis*	4	new	pelagic	0.65	8.6 ± 0.7	2.02	10.0 ± 0.4	−22.1 ± 0.1
*Clinostomus funduloides*	8	new	pelagic	0.64	6.1 ± 0.7	2.06	10.1 ± 0.4	−22.6 ± 0.3
*Nocomis leptocephalus*	8	new	benthic	1.65	13.0 ± 2.3	1.76	9.4 ± 0.4	−23.4 ± 1.3
*Nocomis platyrhynchus*	8	new	benthic	1.47	11.9 ± 4.6	2.21	10.5 ± 0.4	−22.9 ± 0.9
*Notropis rubellus*	8	new	pelagic	0.94	5.6 ± 0.8	2.01	10.0 ± 0.5	−22.5 ± 0.9
*Phenacobius teretulus*	8	new	benthic	0.85	7.1 ± 0.5	2.37	10.9 ± 0.4	−18.3 ± 1.1
*Pimephales notatus*	8	new	pelagic	1.59	7.1 ± 1.0	2.11	10.3 ± 0.2	−23.3 ± 0.6
*Notropis scabriceps*	8	new	pelagic	0.65	5.6 ± 0.4	2.43	11.1 ± 0.2	−22.0 ± 0.4
*Exoglossum laurae*	8	new	benthic	0.92	8.3 ± 1.8	2.36	10.9 ± 0.5	−22.5 ± 1.7
*Campostoma anomalum*	8	new	benthic	3.27	10.6 ± 1.9	2.11	10.3 ± 0.2	−18.7 ± 0.8
*Rhinichthys atratulus*	8	new	benthic	0.88	5.4 ± 0.9	2.50	11.2 ± 0.2	−22.3 ± 0.7
*Cyprinella spiloptera*	5	new	pelagic	0.95	4.9 ± 0.3	1.76	9.4 ± 0.1	−22.1 ± 0.1
*Luxilus coccogenis*	6	Watauga	pelagic	0.60	10.9 ± 0.3	2.41	11.4 ± 0.1	−22.3 ± 0.3
*Notropis leuciodus*	6	Watauga	pelagic	0.65	6.3 ± 0.1	2.38	11.3 ± 0.5	−23.5 ± 0.4
*Rhinichthys atratulus*	6	Watauga	benthic	0.88	6.5 ± 0.2	2.01	10.4 ± 0.6	−22.8 ± 0.2
*Campostoma anomalum*	6	Watauga	benthic	3.27	10.2 ± 0.4	2.04	10.4 ± 0.4	−20.1 ± 0.4
*Clinostomus funduloides*	6	Watauga	pelagic	0.64	7.2 ± 0.1	2.45	11.5 ± 0.4	−21.5 ± 0.5
*Cyprinella galactura*	6	Watauga	pelagic	0.71	11.5 ± 0.3	2.18	10.8 ± 0.4	−23.5 ± 0.7
*Nocomis micropogon*	6	Watauga	benthic	0.91	14.7 ± 0.4	1.95	10.2 ± 0.7	−21.2 ± 0.5
*Semotilus atromaculatus*	4	Watauga	benthic	0.94	10.6 ± 0.4	1.89	10.1 ± 0.3	−23.0 ± 1.2
*Campostoma pauciradii*	5	Halawakee	benthic	3.27	8.1 ± 0.2	2.67	8.1 ± 2.1	−29.6 ± 0.9
*Ericymba amplamala*	4	Halawakee	pelagic	0.79	5.0 ± 0.2	3.46	10.1 ± 1.0	−30.6 ± 1.8
*Semotilus thoreauianus*	5	Halawakee	benthic	1.12	8.0 ± 0.3	1.95	6.3 ± 0.3	−27.8 ± 0.6
*Hybopsis winchelii*	3	Halawakee	benthic	0.80	4.2 ± 0.1	3.04	9.0 ± 0.7	−28.7 ± 0.6
*Cyprinella gibbsi*	5	Hillabee	pelagic	0.69	6.3 ± 0.2	2.08	9.6 ± 0.8	−24.8 ± 0.7
*Campostoma oligolepis*	5	Hillabee	benthic	3.03	9.7 ± 0.4	1.98	9.3 ± 0.2	−20.9 ± 0.5
*Luxilus chrysocephalus*	1	Hillabee	pelagic	0.78	5.7 ± 0.0	1.78	8.9 ± 0.0	−24.8 ± 0.0
*Macrhybopsis aestivalis*	2	Hillabee	benthic	0.76	4.1 ± 0.1	2.43	10.5 ± 0.1	−21.3 ± 0.5
*Notropis baileyi*	5	Hillabee	pelagic	0.97	5.9 ± 0.2	2.18	9.9 ± 2.1	−23.1 ± 1.9
*Notropis stilbius*	5	Hillabee	pelagic	0.66	6.8 ± 0.1	2.47	10.6 ± 0.3	−23.0 ± 0.5
*Phenacobius catastomus*	5	Hillabee	benthic	0.95	8.0 ± 0.2	2.24	10.0 ± 0.3	−21.4 ± 0.3
*Pimephales vigilax*	1	Hillabee	pelagic	1.46	5.5 ± 0.0	2.66	11.1 ± 0.0	−23.8 ± 0.0
*Cyprinella venusta*	5	Uphapee	pelagic	0.76	7.7 ± 0.4	1.67	14.4 ± 1.6	−23.4 ± 0.5
*Ericymba amplamala*	3	Uphapee	pelagic	0.79	5.9 ± 0.3	3.21	18.3 ± 0.0	−21.5 ± 0.5
*Lythrurus bellus*	6	Uphapee	pelagic	0.71	5.0 ± 0.2	1.84	14.8 ± 0.6	−23.7 ± 0.5
*Luxilus chrysocephalus*	6	Uphapee	pelagic	0.78	5.5 ± 0.3	2.87	17.4 ± 0.3	−23.5 ± 0.7
*Macrhybopsis aestivalis*	6	Uphapee	benthic	0.76	4.5 ± 0.1	2.96	17.6 ± 0.4	−24.5 ± 0.6
*Notropis ammophilus*	5	Uphapee	pelagic	1.01	4.6 ± 0.3	2.60	16.7 ± 0.4	−21.5 ± 0.1
*Notropis baileyi*	5	Uphapee	pelagic	0.97	5.0 ± 0.3	2.98	17.7 ± 0.3	−23.4 ± 0.5
*Nocomis leptocephalus*	3	Uphapee	benthic	1.65	5.8 ± 0.5	1.87	14.9 ± 0.3	−22.9 ± 0.8
*Notropis stilbius*	4	Uphapee	pelagic	0.66	6.6 ± 0.3	2.19	15.7 ± 1.0	−23.6 ± 0.8
*Notropis texanus*	5	Uphapee	pelagic	0.72	4.1 ± 0.2	2.89	17.4 ± 0.8	−22.5 ± 0.9
*Notropis uranoscopus*	5	Uphapee	pelagic	0.64	5.0 ± 0.1	2.46	16.4 ± 0.1	−23.6 ± 0.4
*Notropis volucellus*	5	Uphapee	pelagic	0.74	5.2 ± 0.2	2.53	16.6 ± 1.9	−22.0 ± 0.8
*Pimephales vigilax*	3	Uphapee	pelagic	1.46	5.7 ± 0.2	2.98	17.7 ± 0.5	−22.0 ± 0.4
*Macrhybopsis storeriana*	5	Uphapee	benthic	0.89	7.8 ± 0.3	2.22	15.8 ± 0.4	−22.7 ± 0.6
*Campostoma oligolepis*	3	Uphapee	benthic	3.03	6.5 ± 0.4	2.32	15.1 ± 0.7	−26.1 ± 0.3

Only adult specimens (i.e. near maximum body size) were retained for analyses to reduce ontogenetic variation. If present, we also surveyed potentially important production sources: algae (scraped from rock surfaces), terrestrial leaves (representing common riparian species), C_3_ aquatic macrophytes, C_4_ grasses (from the stream bank) and biofilm (generally from the substrate; electronic supplementary material, table S1). Additionally, we sampled invertebrate taxa that are common prey items of benthic and pelagic cyprinids [14]: Ephemeroptera (*Maccafertium* and *Isonychia*), Diptera (Chironomidae), Trichoptera (*Hydropsyche* and Philopotamidae) and Plecoptera (*Acroneuria*), among others (electronic supplementary material, table S1). Cyprinids, prey items and production sources were frozen after collection. In the laboratory, we dissected approximately 5–10 mg of caudal muscle tissue, which was lyophilized until dry (i.e. asymptotic weight), ground into a homogeneous powder and weighed (approx. 1 mg) into tin capsules. Whole invertebrates and subsamples of production sources were prepared in the same way. These materials were analysed for ^15^N and ^13^C at the University of Georgia stable isotope laboratory. Cyprinid isotope values were not corrected for lipid content because their C/N ratios (i.e. less than 3.5) suggest that their lipid content is sufficiently low to prevent biases [16]. Furthermore, cyprinid lipid content (estimated from C/N ratios) was similar among species suggesting uniform effects, if any, from lipids [14]. Fishes were measured from the tip of the snout to the end of the hypural plate (standard length, SL) and were identified using keys [11,15]. Additionally, we dissected the entire digestive tract and calculated relative gut length (RGL) as the linear distance from the oesophagus to the anus divided by SL [17].

We calculated TP using consumer ^15^N signatures such that TP_cyprinid_ = (^15^N_cyprinid_ − ^15^N_base_)/2.5 + 1, where ^15^N_cyprinid_ is the mean ^15^N ratio for a given cyprinid species, ^15^N_base_ is the mean ^15^N ratio among potential invertebrate prey, and 2.5 is a generic fractionation factor derived from meta-analyses [18] that has been confirmed as appropriate for cyprinids by comparing the ^15^N ratios of cyprinids and their gut contents [19]. We compared TP among the five cyprinid assemblages (i.e. sampling sites) using the aov (ANOVA) and Tukey HSD functions in R [20]. We then tested if body size (SL) could predict TP using the lm (linear model) function in R based on both the observed and log-transformed values. We also tested if RGL could predict TP using the lm function; however, the observed RGL measurements were not linearly distributed, so we only used the log-transformed values for this analysis. To further examine the SL–TP relationship, and take advantage of the tendency for cyprinids to be either benthic or pelagic [12], we coded species as either ‘benthic’ or ‘pelagic’ and compared TP, SL and RGL between these two groups using *t*-tests in R. Designations followed Hollingsworth *et al*. [12], except for *N. ammophilus* and *N. amplamala*, which were coded as ‘pelagic’ because they were consistently sampled in association with pelagic species, usually as large mixed species schools or aggregations. Although we restricted sampling to adult specimens, to test the generality of the potential size difference between guilds, we compared the SL of benthic (*n* = 21) and pelagic (*n* = 62) species based on the maximum body sizes reported by Boschung & Mayden [11].

## 3. Results

**Figure 2.**
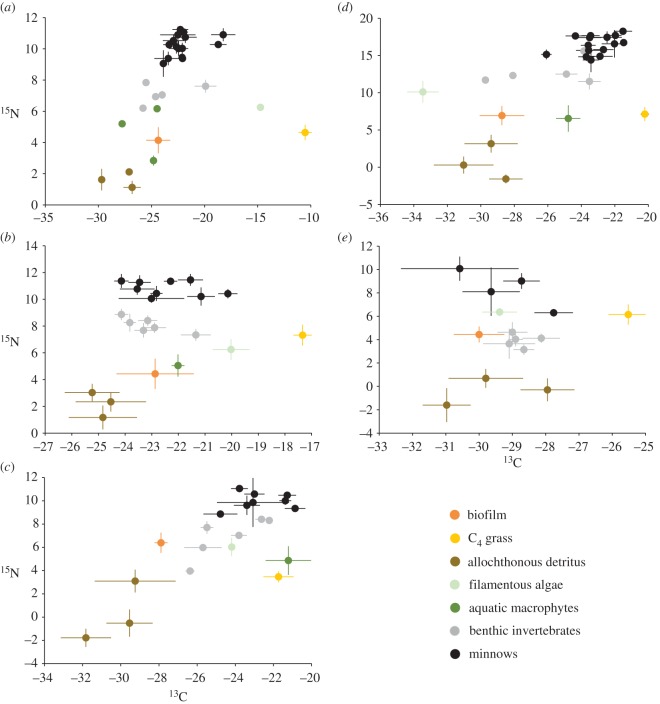
Stable isotope (mean ± s.d.) bi-plots representing the food web structure of five cyprinid assemblages: (*a*) New River, (*b*) Watauga River, (*c*) Hillabee Creek, (*d*) Uphapee Creek and (*e*) Halawakee Creek.

**Figure 3.**
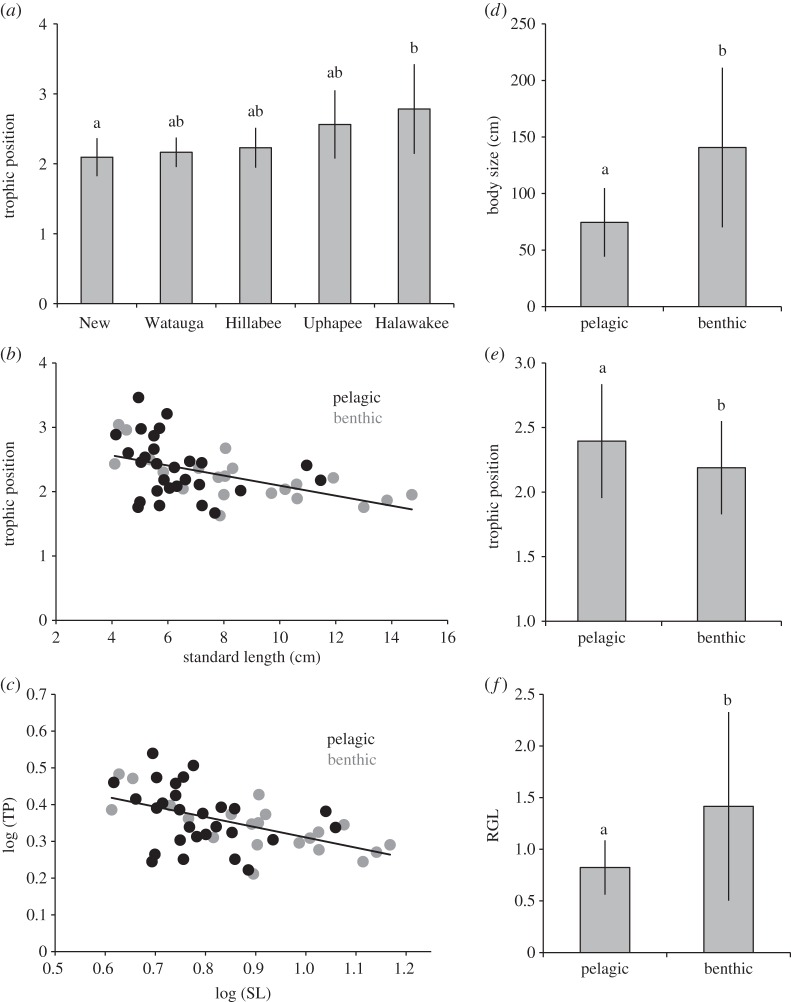
TP among the five cyprinid assemblages (*a*). Relationship between observed (*b*) and log transformed (*c*) body size and TP among cyprinids. Comparison (mean ± s.d.) of maximum SL based on published maximum body sizes (*d*), TP (*e*) and RGL (*f*) between benthic and pelagic cyprinids. Different letters above bars denote statistical significance (*p* < 0.05).

Production sources were well differentiated in the five study streams (electronic supplementary material, table S1; figure 2). Invertebrates were associated with different production sources in each stream based on their ^13^C signatures. For example, they were associated with biofilm in the New and Watauga rivers, with algae in Hillabee Creek, and detritus in Halawakee Creek (figure 2). Cyprinids, however, had stable isotope ratios that were associated with those of invertebrates in all five streams based on ^13^C (figure 2). We sampled a total of 50 populations representing 15 genera and 38 species of cyprinid, 35 species of which are part of the OPM clade (approx. 14% of the OPM clade; table 1) [12]. Cyprinid assemblages (i.e. streams) had different TP (*F *= 3.9, *p *= 0.0074; figure 3*a*). Cyprinids in Halawakee Creek had significantly higher TP than cyprinids in the New River (*p *= 0.019; figure 3*a*). There were also non-significant trends when comparing cyprinid TP in Watauga River and Halawakee Creek (*p *= 0.059) and Uphapee Creek and New River (*p *= 0.055). Cyprinid body size significantly predicted TP using both the observed (*R*^2^ = −0.222, *F*_1,49_ = 13.97, *p *< 0.001; figure 3*b*) and log-transformed (*R*^2^ = −0.254, *F*_1,49_ = 16.66, *p *= 0.00016; figure 3*c*) values such that they had a negative relationship. The smallest cyprinids were approximately half a TP higher in the food chain than the largest cyprinids based on the best-fit line (figure 3*b*). This relationship was also present among benthic species (*R*^2^ = 0.457, *F*_1,21_ = 15.96, *p *< 0.001), but not among pelagic species (*R*^2^ = 0.089, *F*_1,27_ = 2.55, *p *= 0.123). Log-transformed RGL did not significantly predict log-transformed TP (*R*^2^ = 0.0098, *F*_1,49_ = 0.48, *p *= 0.49). Benthic species had, on average, significantly larger observed body sizes than pelagic species (*T *= 3.06, *p *< 0.005). Additionally, maximum body size was, on average, significantly larger than that of pelagic species (*T *= 4.17, *p *= 0.00019; figure 3*d*). Benthic species had significantly lower TP using both observed (*T *= 1.813, *p *= 0.037; figure 3*e*) and log-transformed (*T *= 1.80, *p *= 0.039) values. TP of benthic cyprinids was approximately 0.21 lower than that of pelagic cyprinids (figure 3*e*). Lastly, benthic cyprinids had significantly longer RGL than pelagic cyprinids (*T *= 2.40, *p *= 0.0301; figure 3*f*).

## 4. Discussion

Contrary to the pattern seen across fishes of an increase in TP with body size, we demonstrate that TP is negatively correlated with body size in cyprinids among five diverse assemblages across four major river drainages. This may be due to strongly clade-specific patterns of body size and diet [12] such that the two traits are coevolved with respect to specialization along the benthic–pelagic axis. Several species-poor clades consisting of benthic species such as *Nocomis*, *Campostoma* and *Phenacobius*, among others consume prey directly from, or just above, the substrate and frequently consume biofilm, detritus and algae either intentionally or inadvertently [12]. These materials are depleted in ^15^N relative to invertebrates and consequently higher rates of omnivory results in lower consumer TPs [21]. By contrast, several speciose clades consist of pelagic species such as *Notropis*, *Cyprinella* and *Luxilus*, among others that feed either from the water surface or from the water column via drift [12,14]. Therefore, these species tend to be less omnivorous because they are unlikely to inadvertently consume biofilm, detritus or algae while foraging. Higher degrees of carnivory result in higher TPs among these species [21]. Benthic and pelagic species also tend to be different sizes such that benthic species are larger (figure 3*d*).

Selection pressures that lead to distinct large-bodied benthic and small-bodied pelagic lineages of cyprinids have resulted in body size and TP being coupled during their evolutionary history. The negative relationship between body size and TP is in contrast to general predictions about body size in trophic networks. Body size often increases with TP, either because consumers must eat more nutrient-rich foods to satisfy increased metabolic demands [4] or eat larger prey due to relaxed gape constraints [10]. It is unclear how cyprinids subvert these physiological and mechanical constraints, but we propose that mechanical constraints (i.e. gape) may not be a particularly important limitation because many common invertebrate prey items such as dipterans, ephemeropterans and trichopterans are small [22], and therefore unlikely to impose significant gape limitation among cyprinids. Furthermore, teleost intestines do not produce cellulose to digest plant cell walls [23], and although microorganisms produce these in many teleost guts, such symbiotic production is minimal in cyprinids [24,25]. However, cyprinids use two anatomical modifications to facilitate digestion of plant matter. First, cyprinids have modified pharyngeal arches that assist with prey processing. Mechanical processing (i.e. grinding and tearing) by the pharyngeal arches ruptures plant cell walls prior to digestion [25,26]. Secondly, long digestive tracts increase passage time through the gut thereby increasing exposure to digestive enzymes [17]. In combination, pharyngeal processing and long digestive tracts provide a means for cyprinids to subvert the physiological constraints that generally result in a positive relationship between body size and TP. Indeed, several groups of benthic cyprinids (e.g. *Nocomis* and *Campostoma*) are capable of assimilating nutrients from algae [17,27] and thus may not be particularly nutrient limited by their lower quality food resources. Although benthic cyprinids had longer RGL than pelagic cyprinids, there was not a relationship between RGL and TP. This may be because few cyprinids have intermediate RGL such that cyprinids generally have either poorly developed or well-developed ability to assimilate nutrients from plant material. Additionally, larger cyprinids may avoid metabolic demands by simply eating more, or by saving energy compared with pelagic species by avoiding prolonged swimming in open water [28].

The Cypriniformes as a whole exhibit a weak positive relationship between body size and TP [10]; however, our results suggest that scale (e.g. assemblage, order, global) is important when assessing the relationship between body size and TP. Although there are large-bodied piscivores such as pikeminnows (*Ptychocheilus*) in western North America, most cyprinid assemblages lack representatives from this top trophic level. For example, in eastern North America, where Nearctic cyprinid diversity is concentrated in the OPM clade (more than 250 species), the top predators are drift- or surface-feeding insectivores [11,12]. Therefore, the patterns observed in the four major river basins studied here are likely to be ubiquitous among cyprinids at the assemblage scale. Additionally, negative relationships between body size and TP may occur in many cyprinid assemblages worldwide, because the dominant large-bodied species of cyprinids in Africa and Asia are benthic grazers such as *Labeo*, *Tor* and *Catlocarpio* [29–32]. Our assemblage-based approach is more likely to elucidate local trends than global approaches that pool species into broad taxonomic groups [10], where ecological processes may drive unique relationships among traits. Additionally, global approaches are likely to mask ubiquitous local trends by placing emphasis on taxa that do not ecologically characterize the group, such as large, predatory taxa among cyprinids. Lastly, accessing patterns at various evolutionary and geographical scales may elucidate potential mechanisms that result in associations between body size and TP. For example, some lineages such as *Nocomis* are more diverse in higher latitude temperate streams (i.e. the North Carolina streams) than in lower latitude sub-tropical streams (i.e. the Alabama streams) [11,15], and thus evolutionary history and species distributions are likely to influence the relationship between body size and TP.

## 5. Conclusion

We demonstrate that, contrary to general expectations, there is a negative relationship between body size and TP among cyprinids at the assemblage scale. Our results suggest that scale is important when accessing relationships between body size and TP such that this relationship differs at global and assemblage scales. Highly clade-specific patterns in body size and diet among cyprinids [12,14] have resulted in a counterintuitive negative relationship between these variables. This relationship is probably due to coevolution such that body size and TP are functionally linked. For example, larger bodies are necessary to accommodate the long digestive tracts required by herbivorous and highly omnivorous cyprinids to efficiently breakdown the cellulose-rich foods they consume. Therefore, covariation in these traits probably reflects specialization along the benthic to pelagic axis, which is perhaps the fundamental axis of diversification among fishes [33–36].

## Supplementary Material

Table S1. Data used for analyses in the paper.

## Data accessibility

All data are available in the electronic supplementary material.
