# Supplementary material for: Body size is negatively correlated with trophic position among cyprinids
Source: R Soc Open Sci. 2016 May 11;3(5):150652. doi: 10.1098/rsos.150652 (PMC4892439; doi:10.1098/rsos.150652)
Supplement: Table S1. Data used for analyses in the paper. [file rsos150652supp1.docx]

Table S1. Data used for analyses in the paper. Guild designation, relative gut length (RGL), body size (SL; cm), and trophic position (TP) are only listed for fishes. Full species names are listed for fishes, genera for invertebrates, and generic common names for production sources.

| Sample | *N* | Site | Guild | RGL | SL | TP | ^15^N | ^13^C |
| --- | --- | --- | --- | --- | --- | --- | --- | --- |
| *Rhinichthys cataractae* | 8 | New | Benthic | 0.92 | 5.8±1.7 | 2.30 | 10.8±0.4 | -21.8±1.1 |
| *Luxilus coccogenis* | 3 | New | Pelagic | 0.60 | 7.2±0.3 | 1.78 | 9.5±0.2 | -22.1±0.2 |
| *Semotilis atromaculatus* | 5 | New | Benthic | 0.94 | 7.9±1.9 | 1.63 | 9.1±0.8 | -23.9±0.1 |
| *Notropis photogenis* | 4 | New | Pelagic | 0.65 | 8.6±0.7 | 2.02 | 10.0±0.4 | -22.1±0.1 |
| *Clinostomus funduloides* | 8 | New | Pelagic | 0.64 | 6.1±0.7 | 2.06 | 10.1±0.4 | -22.6±0.3 |
| *Nocomis leptocephalus* | 8 | New | Benthic | 1.65 | 13.0±2.3 | 1.76 | 9.4±0.4 | -23.4±1.3 |
| *Nocomis platyrhynchus* | 8 | New | Benthic | 1.47 | 11.9±4.6 | 2.21 | 10.5±0.4 | -22.9±0.9 |
| *Notropis rubellus* | 8 | New | Pelagic | 0.94 | 5.6±0.8 | 2.01 | 10.0±0.5 | -22.5±0.9 |
| *Phenacobius teretulus* | 8 | New | Benthic | 0.85 | 7.1 | 2.37 | 10.9±0.4 | -18.3±1.1 |
| *Pimephales notatus* | 8 | New | Pelagic | 1.59 | 7.1±1.0 | 2.11 | 10.3±0.2 | -23.3±0.6 |
| *Notropis scabriceps* | 8 | New | Pelagic | 0.65 | 5.6±0.4 | 2.43 | 11.1±0.2 | -22.0±0.4 |
| *Exoglossum laurae* | 8 | New | Benthic | 0.92 | 8.3±1.8 | 2.36 | 10.9±0.5 | -22.5±1.7 |
| *Campostoma anomalum* | 8 | New | Benthic | 3.27 | 10.6±1.9 | 2.11 | 10.3±0.2 | -18.7±0.8 |
| *Rhinichthys atratulus* | 8 | New | Benthic | 0.88 | 5.4±0.9 | 2.50 | 11.2±0.2 | -22.3±0.7 |
| *Cyprinella spiloptera* | 5 | New | Pelagic | 0.95 | 4.9±0.3 | 1.76 | 9.4±0.1 | -22.1±0.1 |
| Hydropsyche | 3 | New |  |  |  |  | 6.9±0.1 | -24.6±0.1 |
| Maccafertium | 3 | New |  |  |  |  | 7.1±0.3 | -24.0±0.1 |
| Isonychia | 3 | New |  |  |  |  | 7.8±0.1 | -25.5±0.1 |
| Elimia | 3 | New |  |  |  |  | 7.6±0.4 | -19.9±1.0 |
| Algae | 3 | New |  |  |  |  | 6.3±0.2 | -14.7±0.0 |
| Biofilm | 3 | New |  |  |  |  | 4.1±0.8 | -24.4±1.1 |
| Red Oak | 3 | New |  |  |  |  | 1.6±0.7 | -29.7±0.1 |
| Yellow Birch | 3 | New |  |  |  |  | 2.1±0.1 | -27.1±0.1 |
| Red Maple | 3 | New |  |  |  |  | 1.1±0.4 | -26.8±0.8 |
| C_4_ Grass | 3 | New |  |  |  |  | 4.6±0.5 | -10.5±0.6 |
| Macrophyte | 3 | New |  |  |  |  | 6.2±0.0 | -24.5±0.2 |
| Macrophyte | 3 | New |  |  |  |  | 5.2±0.1 | -27.8±0.1 |
| Macrophyte | 3 | New |  |  |  |  | 2.8±0.3 | -24.8±0.1 |
| *Luxilus coccogenis* | 6 | Watauga | Pelagic | 0.60 | 10.9±0.3 | 2.41 | 11.4±0.1 | -22.3±0.3 |
| *Notropis leuciodus* | 6 | Watauga | Pelagic | 0.65 | 6.3±0.1 | 2.38 | 11.3±0.5 | -23.5±0.4 |
| *Rhinichthys atratulus* | 6 | Watauga | Benthic | 0.88 | 6.5±0.2 | 2.01 | 10.4±0.6 | -22.8±0.2 |
| *Campostoma anomalum* | 6 | Watauga | Benthic | 3.27 | 10.2±0.4 | 2.04 | 10.4±0.4 | -20.1±0.4 |
| *Clinostomus funduloides* | 6 | Watauga | Pelagic | 0.64 | 7.2±0.1 | 2.45 | 11.5±0.4 | -21.5±0.5 |
| *Cyprinella galactura* | 6 | Watauga | Pelagic | 0.71 | 11.5±0.3 | 2.18 | 10.8±0.4 | -23.5±0.7 |
| *Nocomis micropogon* | 6 | Watauga | Benthic | 0.91 | 14.7±0.4 | 1.95 | 10.2±0.7 | -21.2±0.5 |
| *Semotilus atromaculatus* | 4 | Watauga | Benthic |  | 10.6±0.4 | 1.89 | 10.1±0.3 | -23.0±1.2 |
| Hydropsyche | 3 | Watauga |  |  |  |  | 8.4±0.4 | -23.2±0.4 |
| Maccafertium | 3 | Watauga |  |  |  |  | 7.9±0.4 | -22.9±0.4 |
| Isonychia | 3 | Watauga |  |  |  |  | 7.3±0.4 | -21.3±0.6 |
| Philopotomidae | 3 | Watauga |  |  |  |  | 7.7±0.5 | -23.3±0.3 |
| Simuliidae | 3 | Watauga |  |  |  |  | 8.3±0.7 | -23.8±0.2 |
| Chironomus | 3 | Watauga |  |  |  |  | 8.9±0.4 | -24.1±0.3 |
| Algae | 3 | Watauga |  |  |  |  | 6.3±0.8 | -20.0±0.7 |
| Biofilm | 3 | Watauga |  |  |  |  | 4.4±1.1 | -22.9±1.5 |
| Eastern Sycamore | 3 | Watauga |  |  |  |  | 2.3±0.7 | -24.5±1.3 |
| Red Oak | 3 | Watauga |  |  |  |  | 3.0±0.7 | -25.2±1.0 |
| Red Maple | 3 | Watauga |  |  |  |  | 1.2±0.9 | -24.8±1.3 |
| *Campostoma pauciradii* | 5 | Halawakee | Benthic | 3.27 | 8.1±0.2 | 2.67 | 8.1±2.1 | -29.6±0.9 |
| *Ericymba amplamala* | 4 | Halawakee | Pelagic | 0.79 | 5.0±0.2 | 3.46 | 10.1±1.0 | -30.6±1.8 |
| *Semotilus thoreauianus* | 5 | Halawakee | Benthic | 1.12 | 8.0±0.3 | 1.95 | 6.3±0.3 | -27.8±0.6 |
| *Hybopsis winchelii* | 3 | Halawakee | Benthic | 0.80 | 4.2±0.1 | 3.04 | 9.0±0.7 | -28.7±0.6 |
| Hydropsyche | 3 | Halawakee |  |  |  |  | 3.6±1.3 | -29.1±0.8 |
| Maccafertium | 3 | Halawakee |  |  |  |  | 3.1±0.4 | -28.7±0.3 |
| Philopotomidae | 3 | Halawakee |  |  |  |  | 4.0±0.6 | -28.9±0.2 |
| Acroneuria | 3 | Halawakee |  |  |  |  | 4.6±0.9 | -29.9±0.5 |
| Tipulidae | 3 | Halawakee |  |  |  |  | 4.1±0.4 | -28.1±0.6 |
| Algae | 3 | Halawakee |  |  |  |  | 6.4±0.3 | -29.4±0.5 |
| Biofilm | 3 | Halawakee |  |  |  |  | 4.4±0.7 | -30.0±0.8 |
| C_4_ Grass | 3 | Halawakee |  |  |  |  | 6.1±0.9 | -25.5±0.6 |
| Red Maple | 3 | Halawakee |  |  |  |  | -1.6±1.5 | -31.0±0.7 |
| Red Oak | 3 | Halawakee |  |  |  |  | 0.7±0.8 | -29.8±1.1 |
| Tulip Poplar | 3 | Halawakee |  |  |  |  | -0.3±1.0 | -27.9±0.8 |
| *Cyprinella gibbsi* | 5 | Hillabee | Pelagic | 0.69 | 6.3±0.2 | 2.08 | 9.6±0.8 | -24.8±0.7 |
| *Campostoma oligolepis* | 5 | Hillabee | Benthic | 3.03 | 9.7±0.4 | 1.98 | 9.3±0.2 | -20.9±0.5 |
| *Luxilus chrysocephalus* | 1 | Hillabee | Pelagic | 0.78 | 5.7±0.0 | 1.78 | 8.9±0.0 | -24.8±0.0 |
| *Macrhybopsis aestivalis* | 2 | Hillabee | Benthic | 0.76 | 4.1±0.1 | 2.43 | 10.5±0.1 | -21.3±0.5 |
| *Notropis baileyi* | 5 | Hillabee | Pelagic | 0.97 | 5.9±0.2 | 2.18 | 9.9±2.1 | -23.1±1.9 |
| *Notropis stilbius* | 5 | Hillabee | Pelagic | 0.66 | 6.8±0.1 | 2.47 | 10.6±0.3 | -23.0±0.5 |
| *Phenacobius catastomus* | 5 | Hillabee | Benthic | 0.95 | 8.0±0.2 | 2.24 | 10.0±0.3 | -21.4±0.3 |
| *Pimephales vigilax* | 1 | Hillabee | Pelagic | 1.46 | 5.5±0.0 | 2.66 | 11.1±0.0 | -23.8±0.0 |
| Hydropsyche | 3 | Hillabee |  |  |  |  | 7.0±0.2 | -23.8±0.4 |
| Maccafertium | 3 | Hillabee |  |  |  |  | 5.9±0.2 | -26.0±1.0 |
| Acroneuria | 3 | Hillabee |  |  |  |  | 8.4±0.3 | -22.6±0.3 |
| Tipulidae | 3 | Hillabee |  |  |  |  | 3.9±0.3 | -26.4±0.2 |
| Shrimp | 3 | Hillabee |  |  |  |  | 8.3±0.3 | -22.2±0.1 |
| Algae | 3 | Hillabee |  |  |  |  | 6.0±0.8 | -4.2±0.1 |
| Biofilm | 3 | Hillabee |  |  |  |  | 6.4±0.9 | -27.9±0.3 |
| C_4_ Grass | 3 | Hillabee |  |  |  |  | 3.5±0.8 | -21.7±0.5 |
| Macrophyte | 3 | Hillabee |  |  |  |  | 4.9±1.2 | -21.2±1.2 |
| Eastern Sycamore | 3 | Hillabee |  |  |  |  | 3.1±1.0 | -29.2±2.1 |
| Tulip Poplar | 3 | Hillabee |  |  |  |  | -0.5±1.2 | -29.5±1.2 |
| White Oak | 3 | Hillabee |  |  |  |  | -1.8±0.8 | -31.8±1.3 |
| *Cyprinella venusta* | 5 | Uphapee | Pelagic | 0.76 | 7.7±0.4 | 1.67 | 14.4±1.6 | -23.4±0.5 |
| *Ericymba amplamala* | 3 | Uphapee | Pelagic | 0.79 | 5.9±0.3 | 3.21 | 18.3±0.0 | -21.5±0.5 |
| *Lythrurus bellus* | 6 | Uphapee | Pelagic | 0.71 | 5.0±0.2 | 1.84 | 14.8±0.6 | -23.7±0.5 |
| *Luxilus chrysocephalus* | 6 | Uphapee | Pelagic | 0.78 | 5.5±0.3 | 2.87 | 17.4±0.3 | -23.5±0.7 |
| *Macrhybopsis aestivalis* | 6 | Uphapee | Benthic | 0.76 | 4.5±0.1 | 2.96 | 17.6±0.4 | -24.5±0.6 |
| *Notropis ammophilus* | 5 | Uphapee | Pelagic | 1.01 | 4.6±0.3 | 2.60 | 16.7±0.4 | -21.5±0.1 |
| *Notropis baileyi* | 5 | Uphapee | Pelagic | 0.97 | 5.0±0.3 | 2.98 | 17.7±0.3 | -23.4±0.5 |
| *Nocomis leptocephalus* | 3 | Uphapee | Benthic | 1.65 | 5.8±0.5 | 1.87 | 14.9±0.3 | -22.9±0.8 |
| *Notropis stilbius* | 4 | Uphapee | Pelagic | 0.66 | 6.6±0.3 | 2.19 | 15.7±1.0 | -23.6±0.8 |
| *Notropis texanus* | 5 | Uphapee | Pelagic | 0.72 | 4.1±0.2 | 2.89 | 17.4±0.8 | -22.5±0.9 |
| *Notropis uranoscopus* | 5 | Uphapee | Pelagic | 0.64 | 5.0±0.1 | 2.46 | 16.4±0.1 | -23.6±0.4 |
| *Notropis volucellus* | 5 | Uphapee | Pelagic | 0.74 | 5.2±0.2 | 2.53 | 16.6±1.9 | -22.0±0.8 |
| *Pimephales vigilax* | 3 | Uphapee | Pelagic | 1.46 | 5.7±0.2 | 2.98 | 17.7±0.5 | -22.0±0.4 |
| *Macrhybopsis storeriana* | 5 | Uphapee | Benthic | 0.89 | 7.8±0.3 | 2.22 | 15.8±0.4 | -22.7±0.6 |
| *Campostoma oligolepis* | 3 | Uphapee | Benthic | 3.03 | 6.5±0.4 | 2.32 | 15.1±0.7 | -26.1±0.3 |
| Hydropsyche | 3 | Uphapee |  |  |  |  | 12.3±0.5 | -28.1±0.2 |
| Isonychia | 3 | Uphapee |  |  |  |  | 11.7±0.4 | -29.7±0.2 |
| Philopotomidae | 3 | Uphapee |  |  |  |  | 12.5±0.5 | -24.9±0.7 |
| Acroneuria | 3 | Uphapee |  |  |  |  | 15.6±0.2 | -23.8±0.2 |
| Algae | 3 | Uphapee |  |  |  |  | 10.1±1.5 | -33.5±0.9 |
| Biofilm | 3 | Uphapee |  |  |  |  | 6.9±0.7 | -28.7±1.3 |
| Macrophyte | 3 | Uphapee |  |  |  |  | 6.5±1.8 | -20.2±0.8 |
| C_4_ Grass | 3 | Uphapee |  |  |  |  | 7.1±1.0 | -20.2±0.2 |
